# An Open-Label, Single-Arm, Multicenter, Observational Study Evaluating the Safety and Effectiveness of Akynzeo® in the Management of Chemotherapy-Induced Nausea and Vomiting in India

**DOI:** 10.7759/cureus.56447

**Published:** 2024-03-19

**Authors:** Chandrakanth MV, Boben Thomas, Ghanshyam Biswas, Sumant Gupta, Palanki S Dattatreya, Sagar Bhagat, Saiprasad Patil, Sumit Bhushan, Hanmant Barkate

**Affiliations:** 1 Medical Oncology, Narayana Superspeciality Hospital, Kolkata, IND; 2 Medical Oncology, Caritas Hospital, Kottayam, IND; 3 Medical Oncology, Sparsh Hospital & Critical Care, Bhubaneswar, IND; 4 Medical Oncology, Sarvodaya Hospital & Research Centre, Faridabad, IND; 5 Medical Oncology, Renova Hospitals, Secunderabad, IND; 6 Global Medical Affairs, Glenmark Pharmaceuticals Limited, Mumbai, IND

**Keywords:** mec, hec, vomiting, nausea, nepa, netupitant

## Abstract

Background

Chemotherapy-induced nausea and vomiting is a common and unpleasant treatment-related side effect reported by cancer patients receiving chemotherapy. Akynzeo® or NEPA (NEtupitant + PAlonosetron) is the first fixed combination of netupitant and palonosetron that targets both critical pathways involved in emesis while providing a convenient, single oral dose therapy. The current study aimed to assess the effectiveness and safety of NEPA in a real-world setting in India.

Methodology

This was an open-label, multicenter, prospective, single-arm study conducted at six different locations across India. The study included patients of either gender, aged ≥18 years, naive to chemotherapy, scheduled to receive highly or moderately emetogenic chemotherapy (HEC/MEC), and scheduled to receive oral NEPA, as determined by the investigator.

Results

A total of 360 people were screened and enrolled in the study. HEC was prescribed to 289 (81.64%) patients, while MEC was prescribed to 65 (18.36%) patients. Complete response was achieved in 94.92% of patients during the acute phase, 95.20% during the delayed phase, and 93.22% during the overall phase. During the overall phase, 92.73% and 95.38% of patients on the HEC and MEC regimens, respectively, achieved complete response. Adverse events were reported in 3.88% of patients.

Conclusions

Oral NEPA was found to be effective in the Indian real-world setting, eliciting a >90% complete response with HEC and MEC regimens across the acute, delayed, and overall phases.

## Introduction

Chemotherapy-induced nausea and vomiting (CINV) is a common, unpleasant, treatment-related side effect reported by cancer patients undergoing chemotherapy [1]. CINV may appear before, during, or after chemotherapy administration [2]. If CINV is not controlled appropriately, it can be detrimental to the patients, resulting in dehydration, undernourishment, and electrolyte imbalance. These side effects negatively impact patient’s quality of life and their compliance with treatment [1].

The peripheral 5-hydroxy tryptamine receptor (5-HT3)-related pathway is primarily responsible for acute emesis (within 24 hours of chemotherapy) while the central neurokinin-1 (NK-1) receptor pathway is accountable for delayed emesis (from 25 to 120 hours of chemotherapy) [3]. Advancements in the understanding of CINV pathophysiology and the development of newer anti-emetics have transformed the prevention and treatment of CINV [4]. Akynzeo®, or NEtupitant + PAlonosetron (NEPA), is the first commercially available pharmaceutical oral fixed-dose combination of netupitant, which is a highly selective NK-1 receptor antagonist, and palonosetron, which is a pharmacologically and clinically distinct 5-HT3-receptor antagonist with a long half-life. Akynzeo® targets both the critical pathways involved in emesis and offers a convenient, single, oral dose therapy, thereby improving treatment compliance and CINV control [5]. The safety and efficacy of Akynzeo® were evaluated in three pivotal trials (one phase II and two phase III trials) [6-8]. Akynzeo® was found to be well tolerated and safe when used as prophylaxis for acute and delayed CINV.

A previously conducted multinational study (PrACTICE) with other CINV management drugs reported variation in complete response (CR) (range = 50% to 87%) among patients in different countries. The proportion of patients with no clinically significant nausea was the highest in India, whereas the proportion of patients with no emesis was consistently highest in Australia [9,10]. Thus, there was a considerable variation in the response to the anti-emetic prescribed to patients.

Overall, the management of CINV is complicated, multifactorial, and usually dependent on institutional protocols, thereby creating the clinical need to generate real-world evidence on Akynzeo® use specifically in the Indian setting. Therefore, this prospective, multicenter, real-world study was designed to evaluate the effectiveness and safety of Akynzeo® in a real-world setting in India.

## Materials and methods

Study design

This was an open-label, multicenter, prospective, single-arm study conducted at six centers across India between February 2019 and December 2021. This study was initiated following approval from the institutional ethics committees of individual sites (CTRI/2020/02/023586). The study was carried out following the Declaration of Helsinki and ICH-Good Clinical Practice guidelines. Data confidentiality was maintained throughout the study period. Before participating in the study, each participant provided informed consent.

Patients

This study included patients of either gender aged ≥18 years, naive to chemotherapy, scheduled to receive highly or moderately emetogenic chemotherapy (HEC/MEC), and scheduled to receive oral Akynzeo®, as determined by the investigator. Patients who were scheduled to receive radiation therapy to the abdomen or pelvis within one week of day one or between days one and five were ineligible, as were those who were undergoing bone marrow or stem cell transplants. A history of serious cardiovascular disease or a predisposition to cardiac conduction abnormalities; vomiting, retching, or more than mild nausea within 24 hours of day one; and patients currently enrolled in another clinical trial in which antiemetic treatment was required according to the study protocol were also excluded.

Treatment

All enrolled patients received one capsule of Akynzeo® orally 60 minutes before chemotherapy. Dexamethasone was also allowed in the study at the discretion of the oncologist. Each patient kept a diary from the start of chemotherapy infusion on day one to day six (0-120 hours) of chemotherapy, recording the timing and duration of each emetic episode, the severity of nausea, the use of rescue medication, and any adverse events (AEs).

Endpoints

The primary efficacy endpoint was CR during the overall phase (0-120 hours). Key secondary efficacy endpoints included (a) CR during the acute phase (0-24 hours) and delayed phase (24-120 hours); (b) complete protection during the overall phase (0-120 hours); and (c) severity of nausea was assessed using a Visual Analog Scale (VAS) during the overall phase (0-120 hours). The severity of nausea was evaluated using a 100-mm horizontal VAS ranging from no nausea (of less than 5 mm), mild nausea (≤24 mm), moderate nausea (25-74 mm), severe nausea (75-100 mm), and no significant nausea (NSN) (≤25 mm). The anti-emesis assessment tool was used to assess the impact of CINV on the patient’s daily life. Responses were marked on a 100-mm VAS with anchors of 0 to 10. Patients completed this diary on days two and six.

The primary safety endpoint was also to monitor the occurrence/frequency or incident rate of treatment-emergent adverse events (TEAEs) during the overall phase (0-120 hours). Safety was assessed by recording AEs, TEAEs, serious adverse events (SAEs), physical examinations, vital signs, and urine pregnancy tests (UPTs) during each visit.

Definitions

Overall CR was defined as no vomiting and no need for rescue medication at cycle 1 (time frame: 0-120 hours). CR during the acute phase was defined as no vomiting and no need for rescue medication at cycle 1 (time frame: 0-24 hours). CR during the delayed phase was defined as no vomiting and no need for rescue medication at cycle 1 (time frame: 24-120 hours). Complete control (CC) was defined as no significant (grade 2 and more) nausea, no vomiting, and CR (time frame: 0-120 hours). Complete protection (CP) was defined as no nausea, no vomiting, and no use of rescue medication (time frame: 0-120 hours).

Statistical analysis

A descriptive analysis of demographic characteristics was performed for all 360 patients. Mean, standard deviation, minimum, and maximum were derived for the numerical variables. For categorical variables, the frequency and percentage of the population were presented. A descriptive comparative analysis was performed using the chi-square test, comparing the HEC and MEC groups.

## Results

A total of 360 people were screened and enrolled in the study. For 354 subjects, the data were analyzed descriptively. Six of the enrolled subjects were given low emetogenic chemotherapy agents, so they were excluded from the efficacy analysis. The average age of the patients enrolled in the study was 52.79 ± 11.82 years, with a median of 55 years, and nearly two-thirds (241, 68.08%) of the patients were ≥50 years old. Males made up 35.59% of the total sample, while females made up 64.41% of the sample. The most common types of cancer reported were breast cancer (35.03%) and lung cancer (11.86%). HEC was prescribed to 81.64% (n = 289) of patients, while MEC was prescribed to 18.36% (n = 65) of patients.

Of the 354 patients, 94.92% (n = 336) achieved CR during the acute phase, 95.20% (n = 337) achieved CR during the delayed phase, and 93.22% (n = 330) achieved CR during the overall phase. CR was reported in 92.73% (n = 268) and 95.38% (n = 62) of patients on the HEC and MEC regimens, respectively, during the overall phase (Table 1).

**Table 1:** Complete control among enrolled patients. Complete control rate in acute, delayed, and overall phases among the study participants and those on HEC and MEC regimens. HEC: highly emetogenic chemotherapy; MEC: moderately emetogenic chemotherapy

	Acute (0–24 hours), N (%)	Delayed (24–120 hours), N (%)	Overall (0–120 hours), N (%)
Overall	293 (82.77%)	301 (85.03%)	274 (77.40%)
HEC	240 (83.04%)	245 (84.78%)	224 (77.51%)
MEC	53 (81.54%)	56 (86.15%)	50 (76.92%)

In the overall phase, complete protection and CC were reported in 87.01% (n = 308) and 77.4% (n = 274) of patients, respectively (Tables 2, 3)

**Table 2:** Complete response among enrolled patients. Complete response rate in acute, delayed, and overall phases among the study participants and those on HEC and MEC regimens. HEC: highly emetogenic chemotherapy; MEC: moderately emetogenic chemotherapy

	Acute (0–24 hours), N (%)	Delayed (24–120 hours), N (%)	Overall (0–120 hours), N (%)
Overall	336 (94.92%)	337 (95.20%)	330 (93.22%)
HEC	273 (94.46%)	274 (94.81%)	268 (92.73%)
MEC	63 (96.92%)	63 (96.92%)	62 (95.38%)

**Table 3:** Complete protection among enrolled patients. Complete protection rate in acute, delayed, and overall phases among the study participants and those on HEC and MEC regimens. HEC: highly emetogenic chemotherapy; MEC: moderately emetogenic chemotherapy

	Acute (0–24 hours), N (%)	Delayed (24–120 hours), N (%)	Overall (0–120 hours), N (%)
Overall	320 (90.40%)	322 (90.96%)	308 (87.01%)
HEC	261 (90.31%)	260 (89.97%)	251 (86.85%)
MEC	59 (90.77%)	62 (95.38%)	57 (87.69%)

In the overall phase, NSN (≤25 mm) was reported by 92.38% (n = 327) of patients, while 5.65% (n = 20) had moderate nausea, and 1.98% (n = 7) had severe nausea. Similarly, NSN was reported in 92.04% (n = 266) of HEC patients and 93.84% (n = 61) of MEC patients (Table 4).

**Table 4:** Assessment of the severity of nausea. The severity of nausea among the study participants on HEC and MEC regimens. HEC: highly emetogenic chemotherapy; MEC: moderately emetogenic chemotherapy

Parameter	Overall count (N = 354)	HEC (N = 289)	MEC (N = 65)
No nausea	276 (77.97%)	226 (78.20%)	50 (76.92%)
Mild nausea	51 (14.41%)	40 (13.84%)	11 (16.92%)
Moderate nausea	20 (5.65%)	16 (5.54%)	4 (6.15%)
Severe nausea	7 (1.98%)	7 (2.42%)	0 (0.00%)

Of the total patients enrolled in the study, 14 (3.88%) experienced 20 AEs, including one SAE, where, one patient was hospitalized for a day due to severe vomiting which was not related to the test medication, as per investigators discrete. Pain in the leg (n = 3, 0.83%), abdominal pain, and dyspepsia (n = 2, 0.56% each) were other common AEs reported. Overall, the study medication was well tolerated with no patients discontinuing the drug due to side effects (Table 5).

**Table 5:** Adverse events and serious adverse events among the study participants (n = 360).

Category	N (%)
Serious adverse events	
Acute vomiting	1 (0.28 %)
Adverse events	
Total number of adverse events	19 (5.28%)
Pain in the leg	3 (0.83 %)
Abdominal pain	2 (0.56 %)
Dyspepsia	2 (0.56 %)
General weakness	2 (0.56 %)
Abdominal discomfort	1 (0.28 %)
Dyspnea	1 (0.28 %)
Dysphagia	1 (0.28 %)
Fever with neuropathy	1 (0.28 %)
Hemoptysis	1 (0.28 %)
Loose motion	1 (0.28 %)
Occasional cough	1 (0.28 %)
Oral mucositis	1 (0.28 %)
Pain in the hand	1 (0.28 %)
Tenderness over the right side of the neck	1 (0.28 %)

## Discussion

The current study, which evaluated the effectiveness of NEPA, is the first prospective study conducted among Indian patients in a real-life setting. The NEPA regimen is intended to provide guideline-recommended treatments in a single convenient dose pre-chemotherapy cycle. There is also evidence that NEPA plays a role in receptor internalization and has a synergistic effect on NK-1 inhibition via pathway cross-talk, resulting in long-term inhibition of NK1 and 5-HT3 receptor signaling, and thus contributing to better nausea and vomiting control [11].

This study found that NEPA effectively controlled nausea and vomiting, with CR rates of more than 93% (n = 330) in the overall study population, 92.73% (n = 268) for those on the HEC regimen, and 95.38% (n = 62) for those on the MEC regimen, highlighting sustained control of nausea and vomiting in both acute and delayed phases. Overall CR was higher than in the two landmark trials by Hesketh et al. [8] and Aapro et al. [12] at 89.6% and 74.3%, respectively. Another large-scale real-world study among 2,153 patients receiving HEC and MEC regimens demonstrated 83% CR in the overall phase with NEPA [13]. Importantly, the majority of patients in our study were prescribed HEC regimens, all were chemo-naive, with a median age of 55 years, and two-thirds of the population was female, which were major patient-related risk factors that contribute to the development of CINV. The effectiveness shown in a subset of the high-risk population was an additional input generated by the trial.

Nausea is a significant clinical challenge because it is more bothersome to patients and is more difficult to treat than vomiting, particularly in the delayed phase [14]. This study not only assessed CR (the conventional measure of CINV control) but also assessed CP and CC, the latter of which also takes into account the component of nausea. Overall, 87.01% (n = 308) of patients reported having CP and 77.4 % (n = 272) reported having CC during the overall phase, indicating that nausea was effectively controlled in both cases with oral NEPA. Severity of nausea assessment also reported, NSN (≤25 mm) in 92.38% (n = 327) patients, with 77.97% (n = 276) patients reporting no nausea. The NSN was 60-78% in other real-world studies with NEPA [15,16]. The conventional oral anti-emetic regimen containing aprepitant resulted in no significant nausea in 70-73% of patients, as reported in three randomized controlled trials with cisplatin-based regimens [17-19] and 61% in an anthracycline and cyclophosphamide-based regimen study [20]. Fosaprepitant, an injectable anti-emetic that is also used as a single antiemetic prophylaxis, reported NSN in 71.65% of patients [21].

Overall clinical data from various clinical trials showed that oral NEPA is a well-tolerated drug, which was confirmed in our study. Of the total number of patients prescribed NEPA, 3.88% (n = 14) of patients experienced 20 AEs, with one patient experiencing an SAE in the form of severe vomiting and being hospitalized for a day. Pain in the leg (n = 3), abdominal pain, dyspepsia, and general weakness (n = 2 each) were the most common AEs reported in our study. Real-world studies and landmark clinical trials conducted in the past reported treatment AEs in the range of 5-12%, with constipation, headache, and fatigue as common AEs [7-23].

This study had some limitations that need to be addressed in a large randomized clinical trial in India. The effectiveness was assessed among patients on the first chemotherapy cycle, which has been reported to have the highest risk of CINV; however, due to the study’s design, we were unable to see effectiveness in subsequent cycles. Furthermore, the efficacy in patients with refractory and breakthrough nausea and vomiting was not evaluated. A comparison with the existing standard of care in India will also aid in providing further insights into the effectiveness of NEPA.

## Conclusions

In the Indian real-world setting, oral NEPA was found to be effective, as it prompted a >90% CR across the acute, delayed, and overall phases with the HEC and MEC regimens. Nausea control was also very effective with NEPA. Furthermore, it was also safe and well-tolerated. The single-day oral administration of NEPA will aid in medication and international anti-emetic guideline adherence, as well as adherence to chemotherapy regimens, and may help in the improvement of patients’ quality of life.
