# Correction: An Open-Label, Single-Arm, Multicenter, Observational Study Evaluating the Safety and Effectiveness of Akynzeo® in the Management of Chemotherapy-Induced Nausea and Vomiting in India

**DOI:** 10.7759/cureus.c169

**Published:** 2024-04-19

**Authors:** Chandrakanth MV, Boben Thomas, Ghanshyam Biswas, Sumant Gupta, Palanki S Dattatreya, Sagar Bhagat, Saiprasad Patil, Sumit Bhushan, Hanmant Barkate

**Affiliations:** 1 Medical Oncology, Narayana Superspeciality Hospital, Kolkata, IND; 2 Medical Oncology, Caritas Hospital, Kottayam, IND; 3 Medical Oncology, Sparsh Hospital & Critical Care, Bhubaneswar, IND; 4 Medical Oncology, Sarvodaya Hospital & Research Centre, Faridabad, IND; 5 Medical Oncology, Renova Hospitals, Secunderabad, IND; 6 Global Medical Affairs, Glenmark Pharmaceuticals Limited, Mumbai, IND

This article has been corrected by the Editor-in-Chief to remove first author Sudeep Gupta. Dr. Gupta was erroneously included despite not contributing to the work and not providing consent to be listed as an author. The authors greatly regret this error.

